# Impact of TRMT6 on prognosis and immune microenvironment in ovarian cancer

**DOI:** 10.3389/fonc.2025.1636191

**Published:** 2025-10-30

**Authors:** Jing Zhao, Xiaona Wang, Yazhuo Wang, Na Li

**Affiliations:** ^1^ Department of Gynecology, Hebei General Hospital, Shijiazhuang, China; ^2^ Department of Oncology, Hebei General Hospital, Shijiazhuang, China

**Keywords:** m1A regulator, TRMT6, ovarian cancer, poor prognosis, tumor cells immune escape

## Abstract

**Purpose:**

This study investigates the impact of the m1A regulator TRMT6 on prognosis and the tumor microenvironment in ovarian cancer.

**Methods:**

An analysis of the TCGA database was conducted, supplemented by validation from clinical specimens (13 paired samples), to systematically evaluate the expression characteristics of 10 m1A regulators. The prognostic value was assessed using the Kaplan-Meier Plotter database and Cox regression analysis. Additionally, immunohistochemistry and the Log-rank test were employed to validate the impact of TRMT6 on the prognosis and clinicopathological characteristics of ovarian cancer patients. The ssGSEA algorithm and CIBERSORT were utilized to analyze the influence of TRMT6 on the tumor immune microenvironment. We performed single-gene differential analysis of TRMT6 in the TCGA ovarian cancer database using the DESeq2 package and constructed a ceRNA network.

**Results:**

Three m1A regulators (TRMT10C, TRMT6, YTHDF1) were significantly overexpressed in cancer tissues (p < 0.01). Specifically, among these, TRMT6 and YTHDF1 were significantly associated with lower progression-free survival and overall survival (OS) (p < 0.01). Notably, TRMT6 emerged as an independent prognostic factor for predicting poor overall survival (HR = 2.74; 95% CI, 1.13 - 6.65; P = 0.026). TRMT6 expression had a significant correlation with the pathological stage. Furthermore, TRMT6 expression exhibited a significant negative correlation with eleven tumor-infiltrating immune cell types, including cytotoxic cells (p < 0.01). We also found that in ovarian cancer tissues with high expression of TRMT6, the enrichment scores of T cells gamma delta (p < 0.01) and Mast cells activated (p < 0.05) were significantly lower than those in tissues with low expression. HPSE2 has the most interaction nodes among mRNAs, hsa-miR-17-5p among miRNAs, and Lnc SNHG14 among lncRNAs in the ceRNA network.

**Conclusion:**

The findings suggest that the m1A regulator TRMT6 may drive ovarian cancer progression by promoting immune escape.

## 1 Introduction

**Figure 1:**
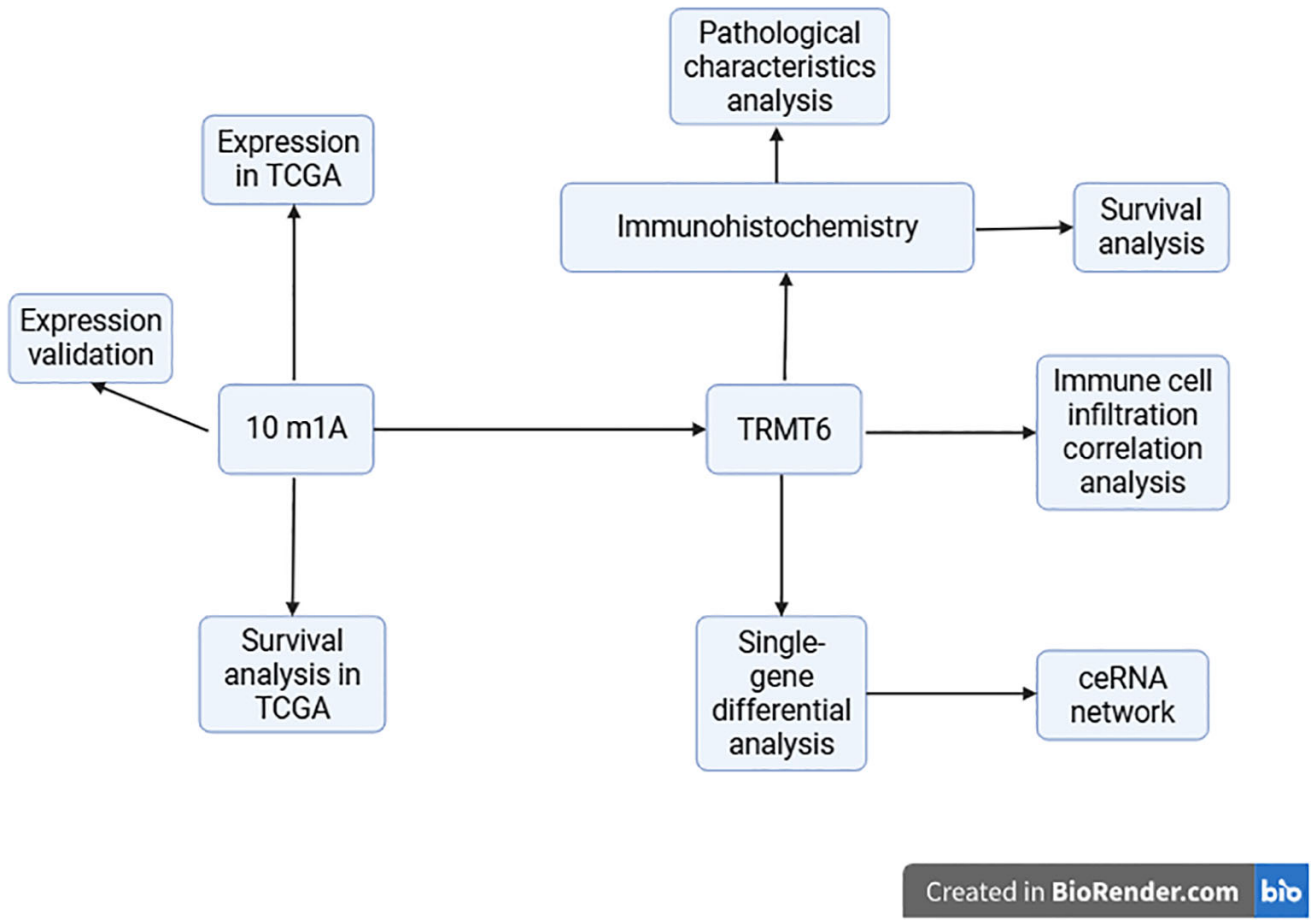
A schematic diagram of the experiment.

Ovarian cancer (OC) is one of the three major gynecological malignancies and the leading cause of death from gynecological tumors (1, 2). It is estimated that in 2025, there will be approximately 20,890 new cases of OC and about 12,730 deaths in the United States (3). In addition to the challenges posed by drug resistance and the absence of individualized targeted therapies, moreover, the heterogeneity of ovarian tumors complicates treatment outcomes. Despite incremental advances in understanding the molecular mechanisms underlying OC, significant gaps remain in our knowledge of post-transcriptional regulation, particularly regarding RNA methylation and its role in tumor progression. RNA methylation encompasses N6-methyladenosine (m6A), N1-methyladenosine (m1A), 5-methylcytosine (m5C), 5-hydroxymethylcytosine (5hmC), and N7-methylguanosine (m7G) (4–6). m1A is linked to various cellular functions, and studies have demonstrated that dysregulation of m1A may be closely associated with tumor proliferation (7), invasion (8), cellular metabolism (9), and the tumor microenvironment (TME) (10). An increasing number of reports suggest that levels of m1A methylation, m1A-related regulators, and m1A-associated RNAs may serve as novel biomarkers for cancer prognosis (11–13). Furthermore, m1A-related regulators and/or m1A modifications on transcripts could represent breakthroughs in cancer treatment (14). In summary, a thorough exploration of the role of m1A regulators in OC is crucial for the development of new prognostic markers and the enhancement of treatment strategies for this disease. Given this, we investigated the role of TRMT6, an m1A regulator, in OC prognosis and the tumor immune microenvironment. The study’s design and findings are shown in **Figure 1**.

## 2 Materials and methods

### 2.1 Data collection

#### 2.1.1 Screening of m1A regulators

Through a review of the published literature, we identified 10 m1A regulators, which include the writers TRMT6, TRMT10C, TRMT61A, and TRMT61B; the readers YTHDC1, YTHDF1, YTHDF2, and YTHDF3; and the erasers ALKBH1 and ALKBH3 (15–17).

#### 2.1.2 UCSC XENA

To analyze the expression of m1A regulators in OC tissues, RNA sequencing data in TPM format was obtained from The Cancer Genome Atlas (TCGA) and the Genotype-Tissue Expression (GTEx) project. This data has been uniformly processed through the Toil pipeline and is accessible via the UCSC XENA database (https://xenabrowser.net/datapages/). The dataset includes 88 normal tissues from GTEx and 427 OC tissues from TCGA (18). For data processing, a log2(value + 1) transformation was applied, and no filtering strategy was implemented.

#### 2.1.3 Kaplan–Meier plotter

We utilized the Kaplan-Meier plotter online database (https://kmplot.com/analysis/index.php?p=service&cancer=ovar) to investigate the prognostic value of m1A regulators in OC patients. The hazard ratio (HR), 95% confidence interval (CI), and log-rank p-value were clearly presented.

#### 2.1.4 TCGA

We downloaded and organized the RNA sequencing (RNAseq) data from the TCGA-OV project, which was processed through the STAR pipeline, from the TCGA database (https://portal.gdc.cancer.gov). We extracted the data in Transcripts Per Million (TPM) format along with clinical data. The data filtering strategy involved the removal of normal samples. The data processing method applied was log2(value + 1). The analysis was conducted using R (version 4.2.1), with the circlize package (version 0.4.1) employed for visualization. The processing steps included analyzing the correlation between pairwise variables in the dataset and visualizing the correlation results using the circlize package. The statistical method utilized for this analysis was Spearman correlation. We employed the Wilcoxon rank sum test (Mann-Whitney U test) as our statistical method for comparing two independent groups. R packages: ggplot2[3.4.4], stats[4.2.1], car[3.1-0].

#### 2.1.5 Tissue collection

Between January 2023 and May 2023, a total of 13 cases of OC tissues were collected from Hebei General Hospital. This cohort comprised 3 cases of high-grade serous OC, 3 cases of ovarian endometrioid adenocarcinoma, 3 cases of ovarian mucinous adenocarcinoma, and 4 cases of ovarian granulosa cell tumour, alongside 13 cases of normal ovarian tissues obtained through surgical resection. The study received approval from the Ethics Committee of Hebei Provincial People’s Hospital (approval number: 2023047), and informed consent was secured from all participating patients. The collected tissue samples were promptly placed in liquid nitrogen and subsequently transported to a -80°C freezer for storage in preparation for RNA extraction. Additionally, from January 2015 to May 2025, 92 cases of OC tissues, which had been embedded in paraffin post-surgical resection, were utilized for immunohistochemical experiments. This group included 40 cases of high-grade serous OC, 1 case of ovarian granulosa cell tumour, 5 cases of ovarian endometrioid adenocarcinoma, 6 cases of ovarian mucinous adenocarcinoma, 4 cases of ovarian clear cell carcinoma, and 1 case of low-grade serous OC, along with 35 cases of normal ovarian tissues. The study was also approved by the Ethics Committee of Hebei Provincial People’s Hospital (approval number: 2022124), with informed consent obtained from all patients involved.

### 2.2 Real-time quantitative PCR

Total RNA was extracted using TRIzol reagent (Tiangen, Beijing, China). cDNA was obtained using the FastQuant First-Strand cDNA Synthesis Kit (Tiangen, Beijing, China), and qPCR was performed on a thermal cycler (ABI, USA, 7500) using the SYBR Green PCR Kit (Tiangen, Beijing, China) to detect gene expression. The reaction conditions were as follows: 95°C for 15 minutes, 95°C for 10 seconds, 60°C for 20 seconds, and 72°C for 32 seconds, for a total of 40 cycles. β-actin was used as the internal reference. All data were analyzed using the 2^-ΔΔCt^ method. Primer sequences are listed in **Table 1**.

**Table 1:** Primer sequence for qRT-PCR.

Primer sequence for qRT-PCR
TRMT10C
F: 5’-TCAAGCTGCTAGAAACCACTG-3’
R: 5’-TCTGTGCAAAGCACCATCTATT-3’
TRMT61B
F: 5’- TTCGACCTCGGTAGCGGACT-3’ R: 5’- AGTCCCGTTCGGCAAGATCG-3’
TRMT6
F: 5’-GGTGCTGAAACGTGAAGATGT-3’
R: 5’-CTTGGGCTGTAGACTTCCTCC-3’
TRMT61A
F: 5’-GCCTTCGTCCACTCATGTCCAAG-3’
R: 5’-CCACTCTGCCGCTCCTCTCC-3’
ALKBH3
F: 5’-TACCACTGCTAAGAGCCATCTCC-3’
R: 5’-GACAGGCTGATTTCATACACACC-3’
ALKBH1
F: 5’-GCTGAAGCAGGGATCCTGAA-3’
R: 5’-CGGACTGTCCAAAGCTGAATG-3’
YTHDC1
F: 5’-ATCTTCCGTTCGTGCTGTCC-3’
R: 5’-GGACCATACACCCTTCGCTT-3’
YTHDF1
F: 5’-ACCTGTCCAGCTATTACCCG-3’
R: 5’-TGGTGAGGTATGGAATCGGAG-3’
YTHDF2
F: 5’-TAGCCAACTGCGACACATTC-3’
R: 5’-CACGACCTTGACGTTCCTTT-3’
YTHDF3
F: 5’-TGTTGTGGACTATAATGCGTATGC-3’
R: 5’-AAGCGAATATGCCGTAATTGGTTA-3’
β-actin
F: 5’-GGCACCACACCTTCTACAATGAC-3’
R: 5’-GGATAGCACAGCCTGGATAGCA-3’

### 2.3 Immunohistochemistry

Paraffin sections were prepared for immunohistochemical staining. Following routine dewaxing and hydration, the sections were treated with fresh 0.3% methanol-hydrogen peroxide for blocking. Antigen retrieval was conducted using citrate buffer, and the sections were incubated overnight at 4°C with TRMT6-specific antibodies. After washing with PBS, the sections were incubated with the corresponding secondary antibodies at 37°C for 15 minutes. Following DAB development, the sections were counterstained, dehydrated, cleared, and mounted. Five random fields of view were selected from each section and observed under an OLYMPUS BX41TF (Japan) optical microscope at ×400 magnification. Three researchers, blinded to the clinical features and outcomes, independently examined and scored the sections. The expression of TRMT6 was quantified by multiplying the average staining intensity (ranging from 0 to 3: 0 indicates no staining; 1 indicates mild staining; 2 indicates moderate staining; 3 indicates intense staining) by the percentage of positive staining (ranging from 0 to 4: 0 indicates 0%; 1 indicates 0%-25%; 2 indicates 26%-50%; 3 indicates 51%-75%; 4 indicates 76%-100%). The final score was derived from the average of the scores calculated by the three researchers, with a score greater than 6 considered positive.

### 2.4 Survival curve analysis

We employed the COX regression analysis and Log-rank test to examine the correlation between TRMT6 expression and the prognosis in OC. When the variables do not satisfy the proportional hazards assumption, the Log-rank test is selected; otherwise, the COX regression analysis is used.

### 2.5 Pathological characteristics analysis

We examined the correlation between TRMT6 expression and pathological characteristics such as pathological staging, grading, lymph node metastasis, Omental metastasis, the levels of CA125 and HE4 in the blood by Fisher test or T-test.

### 2.6 Correlation analysis between TRMT6 expression and tumor-infiltrating immune cells

Download and organize RNAseq data from the STAR pipeline of the TCGA-OV (Ovarian Serous Cystadenocarcinoma) project from the TCGA database, and extract data in TPM format as well as clinical data. Based on the ssGSEA algorithm provided in the R package GSVA [1.46.0] (19), the immune infiltration of the corresponding cloud data was calculated using the markers of 24 immune cells provided in Bindea G’s article (20). Based on the core algorithm of CIBERSORT (analyzed by the CIBERSORT.R script), the markers of 22 immune cells provided by the CIBERSORTx website (https://cibersortx.stanford.edu/) were utilized to calculate the immune infiltration of the uploaded data (21, 22).

### 2.7 Differential gene expression analysis

We used the DESeq2 package to find differentially expressed genes linked to high versus low TRMT6 expression in the TCGA ovarian cancer database, with these cutoffs: FDR < 0.05 and |logFC| ≥ 2.5.

### 2.8 Construction of the ceRNA network

To better explore the regulatory mechanism of TRMT6 in ovarian cancer, we focused on TRMT6 as a key gene and performed single-gene differential analysis using the TCGA ovarian cancer database, which identified eight DEGs (PRLHR, NKX2-1, ZIC3, DPYSL5, HPSE2, ST8SIA3, VSTM2B, and BTBD17). Using the miRDB and Starbase online databases, we found 76 miRNAs and 243 lncRNAs. The ceRNA network was visualized by using Cytoscape software.

### 2.9 Statistical methods

Quantitative data processing and analysis were conducted using Opticon Monitor software (version 3.1). The ΔCT value was calculated as the difference between the CT value of the target gene and the CT value of β-actin. Subsequently, the relative corrected value, ΔΔCT, was computed for all samples, allowing for the determination of the relative expression level of the target gene using the formula: Relative quantity of the target gene = 2^^-ΔΔCT^. The statistical methods employed in this study are as follows: For numeric variables, if the data adhere to a normal distribution and pass the homogeneity of variance test, the T-test is utilized for comparing two groups. In cases where the data meet the normal distribution criteria but fail the homogeneity of variance test, the Welch t-test is applied. Conversely, if the data do not conform to a normal distribution, the Wilcoxon test is employed for group comparisons. For categorical variables, when all expected frequencies exceed 5 and the total sample size is greater than or equal to 40, the Chi-square test is used for intergroup comparisons. If the expected frequencies range between 1 and 5, and the total sample size is at least 40, the continuity corrected Chi-square test (Yates’ correction) is applied. In instances where expected frequencies are less than 1 or the total sample size is below 40, Fisher’s exact test is utilized for intergroup comparison. Additionally, correlation analysis of immune infiltration was conducted using Spearman’s rank correlation coefficient. The analysis was performed using R software (version 4.2.1), involving the R packages ggplot2 [3.4.4], stats [4.2.1], car [3.1-0], survival [3.3.1], and survminer [0.4.9]. Additionally, SPSS version 19.0 was utilized for statistical analysis. All statistical tests were two-tailed, with a significance level set at P < 0.05.

## 3 Results

### 3.1 Expression of 10 m1A regulators in OC and normal ovarian tissues

**Figure 2:**
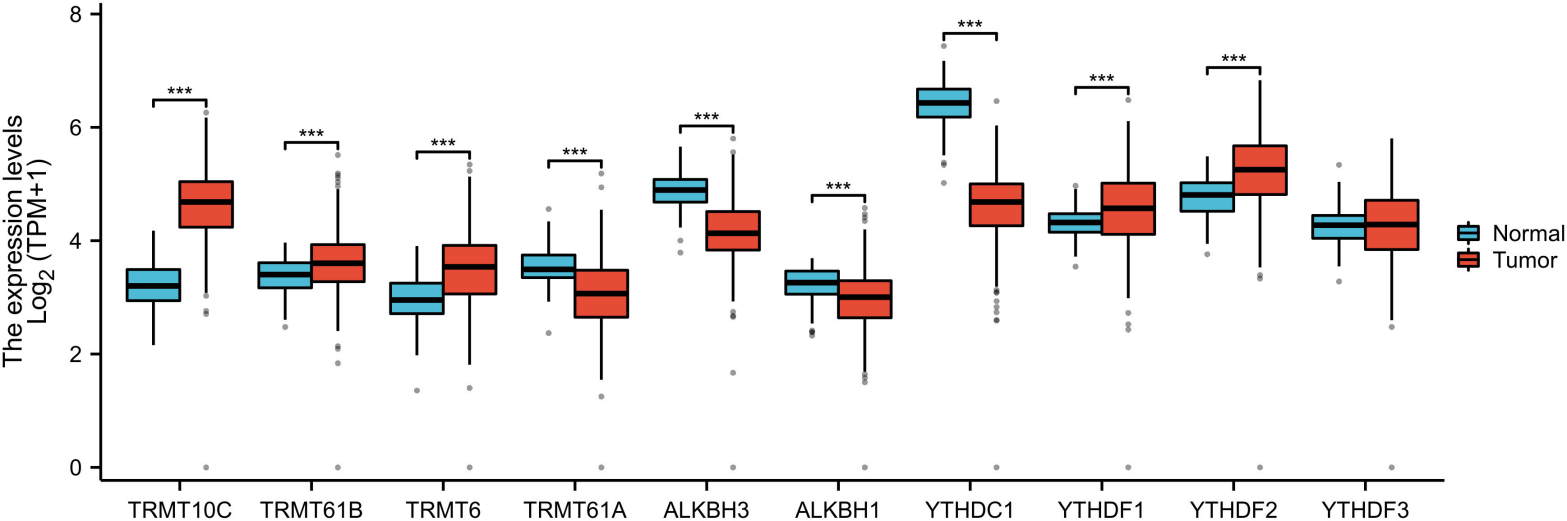
The differential expressions of 10 m1A regulators between ovarian cancer and normal tissues from the TCGA-GTEX database. “***” means <0.001.

**Figure 3:**
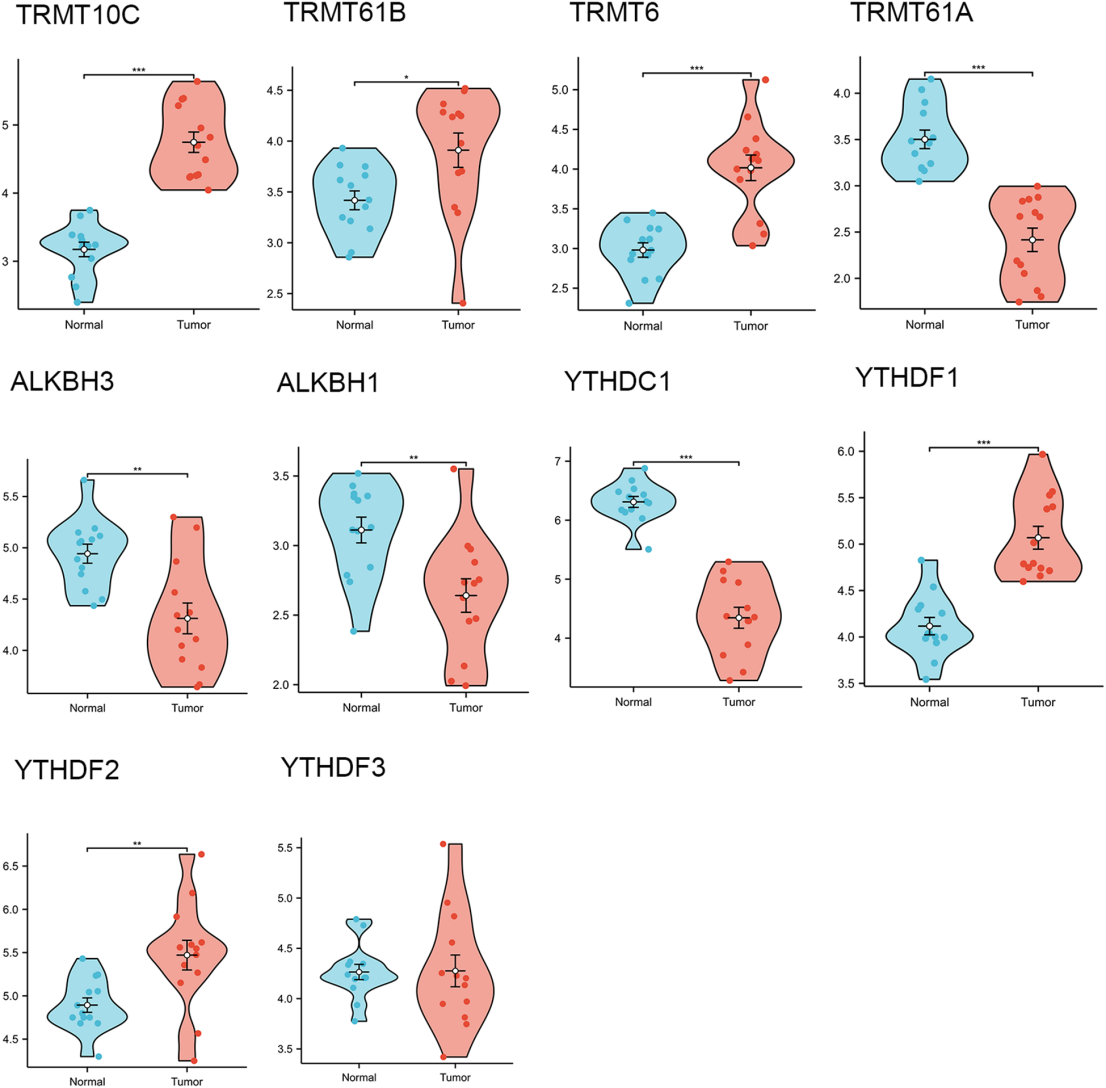
The differential expressions of 10 m1A regulators in ovarian cancer tissues (n=13) and normal tissues (n=13) collected in our hospital. “*” means <0.05, “**” means <0.01, “***” means <0.001.

Through a comprehensive review of the published literature, we identified ten m1A genes, namely TRMT10C, TRMT61B, TRMT6, TRMT61A, ALKBH3, ALKBH1, YTHDC1, YTHDF1, YTHDF2, and YTHDF3. In the TCGA-GTEx-OV RNAseq TOIL TPM dataset, we observed that, except for YTHDF3, the remaining nine m1A regulators exhibited statistically significant differences in expression between OC tissues and normal tissues. Specifically, the expression levels of TRMT10C, TRMT61B, TRMT6, YTHDF1, and YTHDF2 were significantly higher in OC tissues compared to normal tissues, whereas the other four regulators showed elevated expression in normal tissues relative to OC tissues (**Figure 2**). Furthermore, quantitative reverse transcription polymerase chain reaction (qRT-PCR) analysis conducted on 13 OC tissues and 13 normal ovarian tissues confirmed that, apart from YTHDF3, the other nine m1A regulators displayed statistically significant differences in expression levels between OC and normal tissues. Among these, TRMT10C, TRMT61B, TRMT6, YTHDF1, and YTHDF2 exhibited higher expression in OC tissues than in normal tissues, while the remaining four regulators had higher expression in normal tissues compared to OC tissues (**Figure 3**), corroborating the expression differences observed in the database.

### 3.2 The impact of m1A regulators’ expression on the prognosis of OC patients

**Figure 4:**
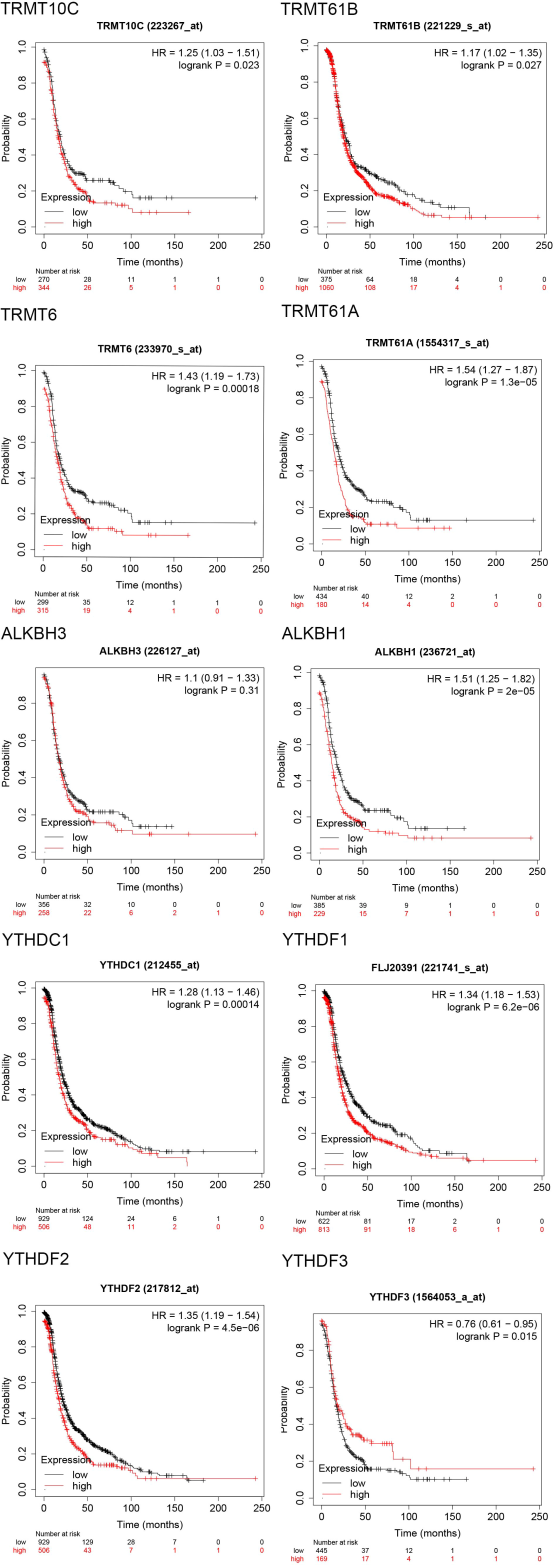
Progression free survival (PFS) curves of 10 m1A regulators in the Kaplan-Meier Plotter database.

**Figure 5:**
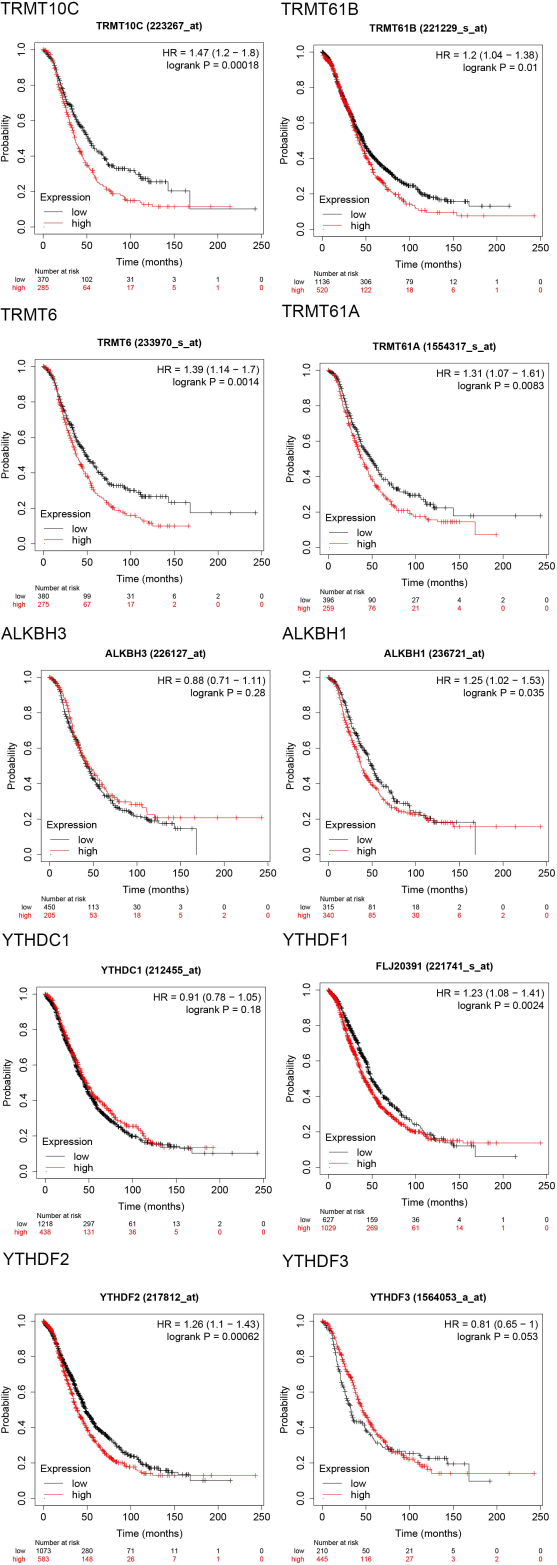
Overall survival (OS) curves of 10 m1A regulators in the Kaplan-Meier Plotter database.

The progression-free survival (PFS) curves derived from the Kaplan-Meier Plotter database (**Figure 4**) indicate that high expression levels of eight m1A regulators are associated with reduced PFS. These regulators include TRMT10C (HR = 1.25; 95% CI, 1.03-1.51; P = 0.023), TRMT61B (HR = 1.17; 95% CI, 1.02-1.35; P = 0.027), TRMT6 (HR = 1.43; 95% CI, 1.19-1.73; P = 0.00018), TRMT61A (HR = 1.54; 95% CI, 1.27-1.87; P = 1.3e-05), ALKBH1 (HR = 1.51; 95% CI, 1.25-1.82; P = 2e-05), YTHDC1 (HR = 1.28; 95% CI, 1.13-1.46; P = 0.00014), YTHDF1/FLJ20391 (HR = 1.34; 95% CI, 1.18-1.53; P = 6.2e-06), and YTHDF2 (HR = 1.35; 95% CI, 1.19-1.54; P = 4.5e-06). In contrast, high expression of YTHDF3 (HR = 0.76; 95% CI, 0.61-0.95; P = 0.015) correlates with improved progression-free survival, with all differences being statistically significant. Furthermore, the overall survival (OS) curve obtained from the Kaplan-Meier Plotter database (**Figure 5**) reveals that high expression of seven m1A regulators is associated with decreased OS. These regulators include TRMT10C (HR = 1.47; 95% CI, 1.2-1.8; P = 0.00018), TRMT61B (HR = 1.2; 95% CI, 1.04-1.38; P = 0.01), TRMT6 (HR = 1.39; 95% CI, 1.14-1.7; P = 0.0014), TRMT61A (HR = 1.31; 95% CI, 1.07-1.61; P = 0.0083), ALKBH1 (HR = 1.25; 95% CI, 1.02-1.53; P = 0.035), YTHDF1/FLJ20391 (HR = 1.23; 95% CI, 1.08-1.41; P = 0.0024), and YTHDF2 (HR = 1.26; 95% CI, 1.1-1.43; P = 0.00062).

### 3.3 Immunohistochemical validation of TRMT6 expression in OC tissues and normal tissues

**Figure 6:**
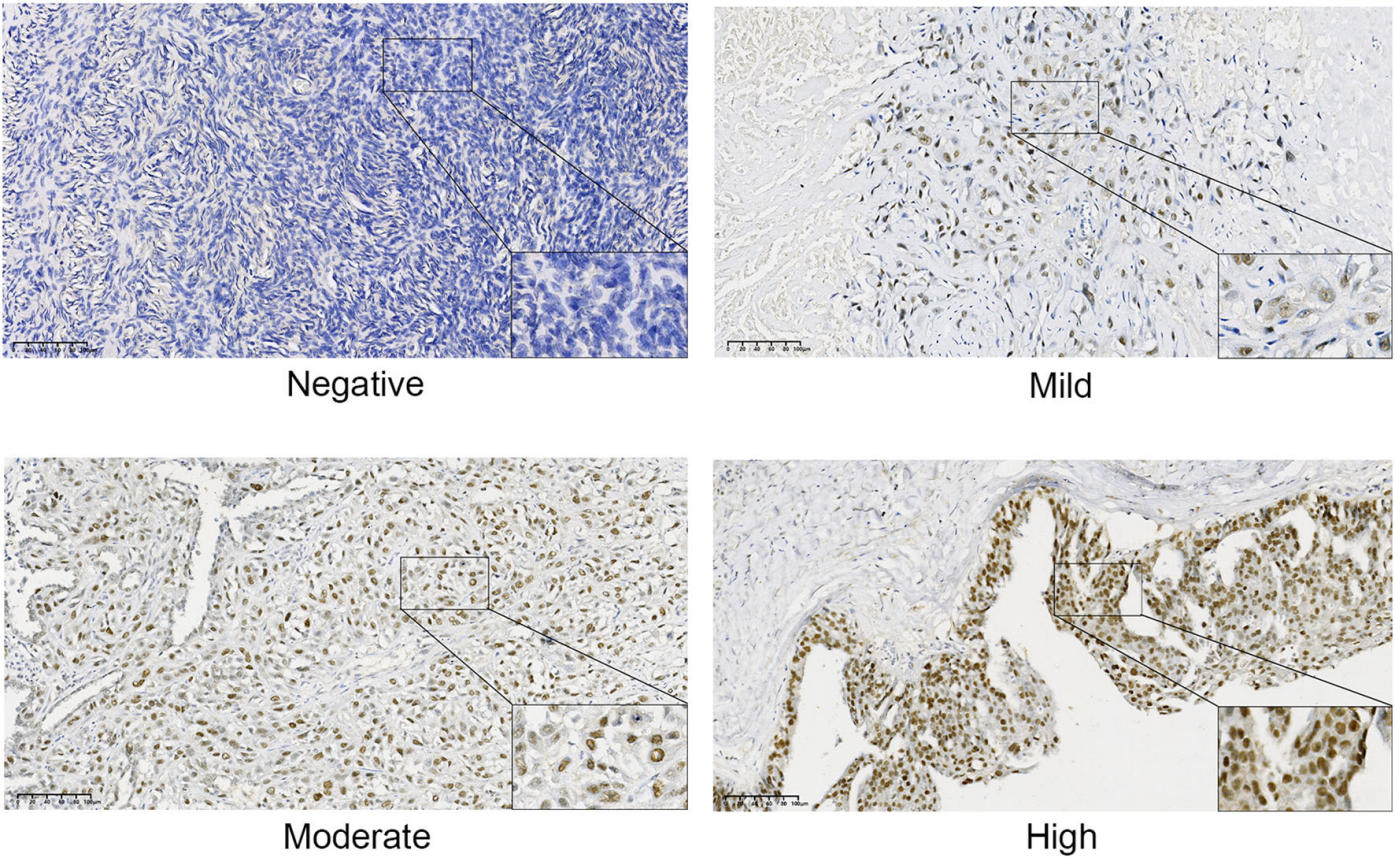
Immunohistochemical staining results of TRMT6 expression in ovarian cancer and normal tissues.

The immunohistochemical results, as illustrated in **Figure 6**, indicated that TRMT6 was positively expressed in 38 of the 57 OC tissues, whereas only 6 of the 35 normal ovarian tissues exhibited positive expression, with the remainder being negative. This difference was statistically significant (P < 0.001).

### 3.4 The relationship between TRMT6 expression and overall survival in OC patients

**Figure 7:**
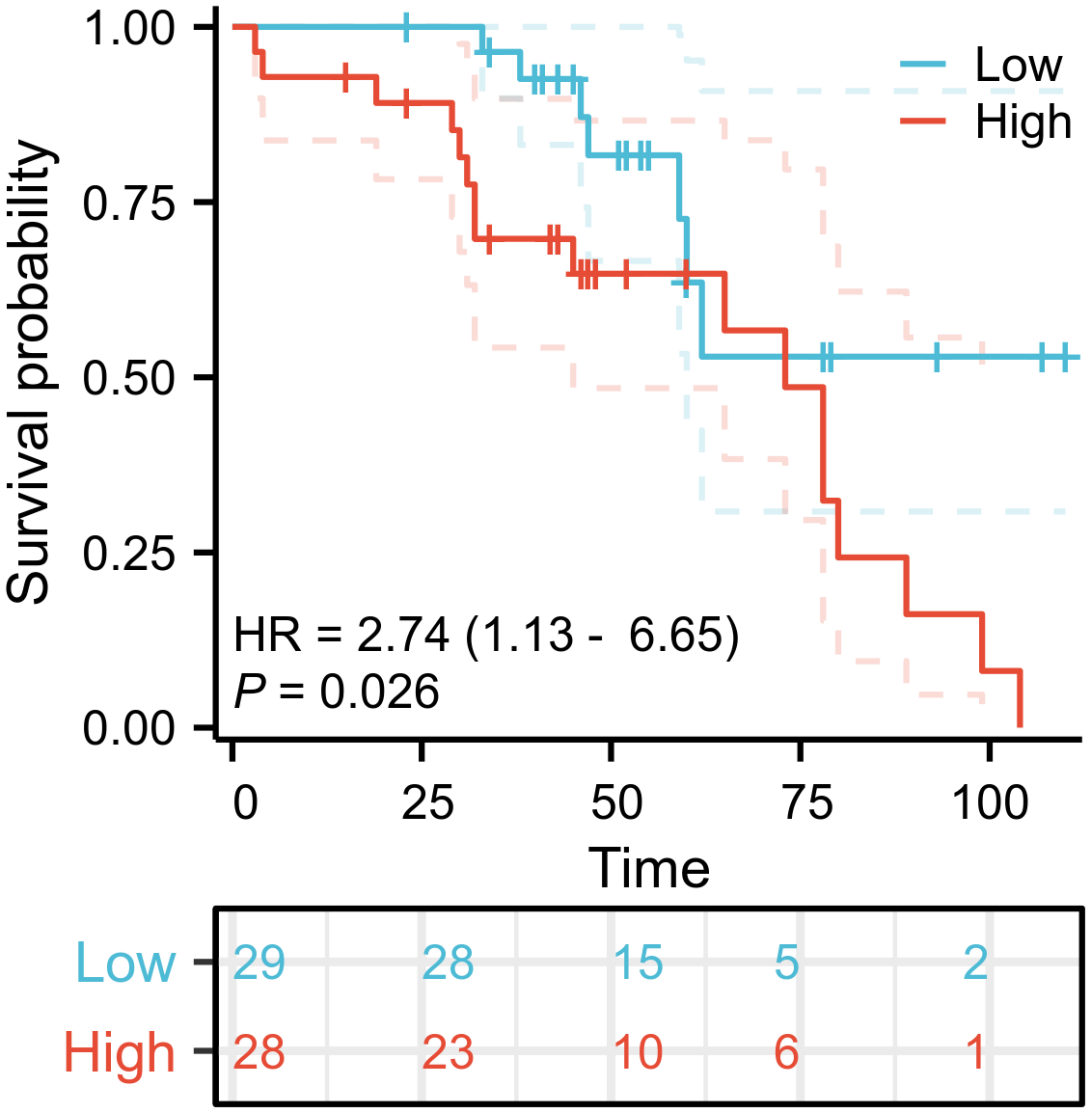
Overall survival (OS) curves of TRMT6 in ovarian cancer patients.

Cox regression analysis indicated that patients exhibiting high TRMT6 expression in OC tissues had a significantly lower overall survival rate compared to those with low TRMT6 expression (HR = 2.74; 95% CI, 1.13-6.65; P = 0.026). The difference was statistically significant, as illustrated in **Figure 7**.

### 3.5 Correlation analysis of TRMT6 expression with clinicopathological characteristics of OC patients

There was no statistically significant correlation between TRMT6 expression and various clinicopathological characteristics in OC patients. These characteristics included pathological stage (p=0.022), age (p=0.492), lymphatic invasion (p=0.483), Omental invasion (p=0.516), primary therapy outcome (p=0.313), pathological type (p=0.338), and whether the cancer was unilateral or bilateral (p=0.196), as illustrated in **Table 2**.

**Table 2:** Correlation of the expression levels of TRMT6 with the clinicopathological characteristics of ovarian cancer patients.

Characteristics	Score in IHC <6.5	Score in IHC >6.5	P value
n	28	29	
Pathological.Stage, n (%)			0.022
Stage III	9 (32.1%)	17 (58.6%)	
Stage IV	2 (7.1%)	4 (13.8%)	
Stage I	8 (28.6%)	7 (24.1%)	
Stage II	9 (32.1%)	1 (3.4%)	
Age, mean ± sd	54.571 ± 10.119	56.621 ± 12.123	0.492
Lymphatic invasion, n (%)			0.483
Yes	10 (35.7%)	13 (44.8%)	
No	18 (64.3%)	16 (55.2%)	
Omental invasion, n (%)			0.516
Yes	12 (42.9%)	10 (34.5%)	
No	16 (57.1%)	19 (65.5%)	
Primary therapy outcome, n (%)			0.313
PD	2 (7.1%)	3 (10.3%)	
PR	5 (17.9%)	11 (37.9%)	
SD	2 (7.1%)	2 (6.9%)	
CR	19 (67.9%)	13 (44.8%)	
Pathologic.Type, n (%)			0.338
high-grade serous ovarian cancer	17 (60.7%)	23 (79.3%)	
granulosa cell tumor	1 (3.6%)	0 (0%)	
ovarian endometrioid carcinoma	4 (14.3%)	1 (3.4%)	
mucinous ovarian cancer	3 (10.7%)	3 (10.3%)	
ovarian clear cell carcinoma	3 (10.7%)	1 (3.4%)	
low-grade serous ovarian cancer	0 (0%)	1 (3.4%)	
Unilateral or bilateral, n (%)			0.196
Bilateral	7 (25%)	12 (41.4%)	
Right	12 (42.9%)	13 (44.8%)	
Left	9 (32.1%)	4 (13.8%)	
CA125, n (%)			0.851
Yes	19 (67.9%)	19 (65.5%)	
No	9 (32.1%)	10 (34.5%)	
HE4, n (%)			0.514
Yes	14 (50%)	17 (58.6%)	
No	14 (50%)	12 (41.4%)	

### 3.6 Correlation analysis between TRMT6 expression and immune cell infiltration in OC

**Figure 8:**
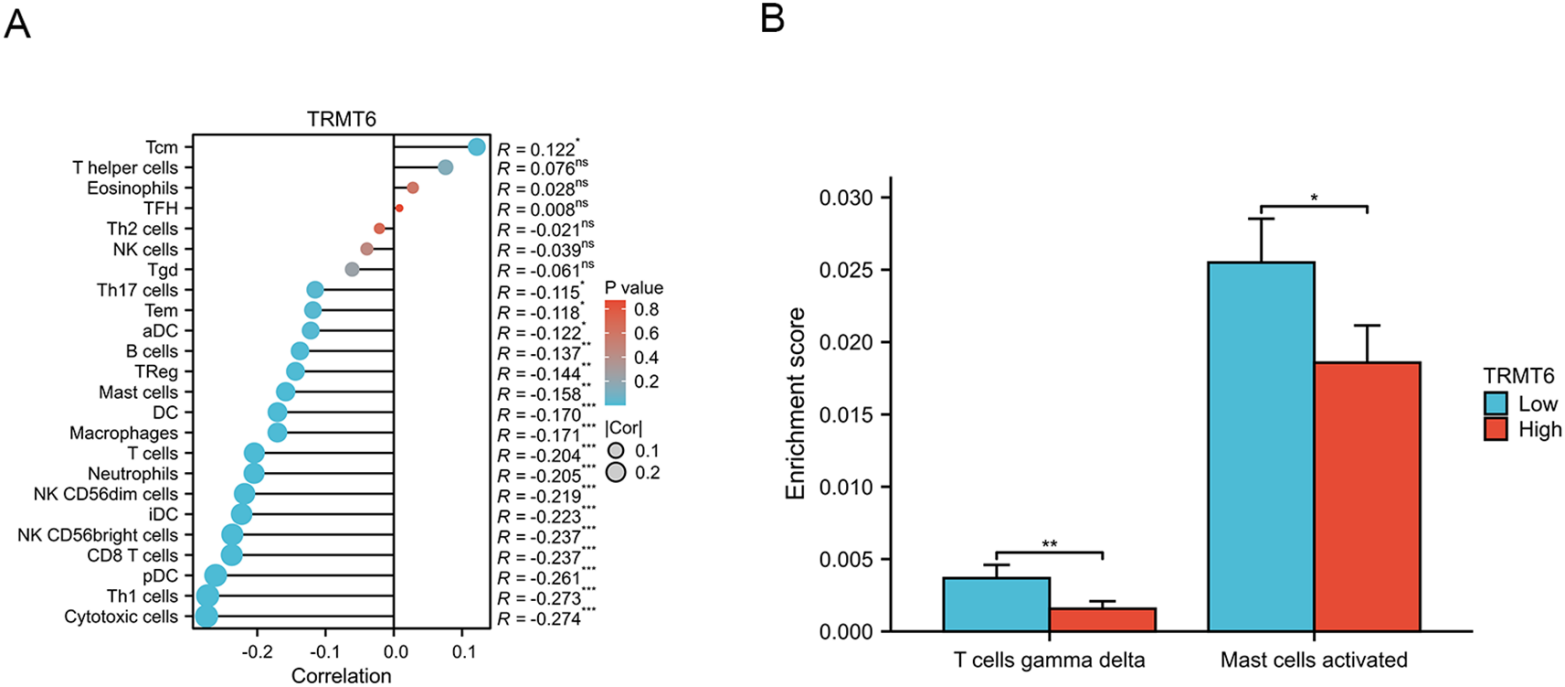
Correlation analysis between TRMT6 expression and tumor immune infiltration **(A)** The lollipop charts **(B)** The differential results of immune cell enrichment scores between high and low TRMT6 expression groups in ovarian cancer tissues from the TCGA database. “*” means <0.05, “**” means <0.01, “***” means <0.001.

The expression of TRMT6 exhibits a weak negative correlation with the infiltration of various immune cell types in TCGA-OV/RNAseq/STAR/TPM dataset (**Figure 8A**), including cytotoxic cells (R=-0.274, p=5.3e-08), Th1 cells (R=-0.273, p=6.3e-08), pDC cells (R=-0.261, p=2.4e-07), CD8T cells (R=-0.237, p=2.8e-06), NK CD56 bright cells (R=-0.237, p=3e-06), iDC cells (R=-0.223, p=1.1e-05), NK CD56 dim cells (R=-0.219, p=1.7e-05), Neutrophils (R=-0.205, p=5.7e-05), T cells (R=-0.204, p=5.8e-05), Macrophages (R=-0.171, p=0.00083) and DC cells (R=-0.170, p=0.00084). Furthermore, we also found that in ovarian cancer tissues with high expression of TRMT6, the enrichment scores of T cells gamma delta(p<0.01)and Mast cells activated (p<0.05) were significantly lower than those in tissues with low expression (**Figure 8B**).

### 3.7 Construction of a competing endogenous RNA network

**Figure 9:**
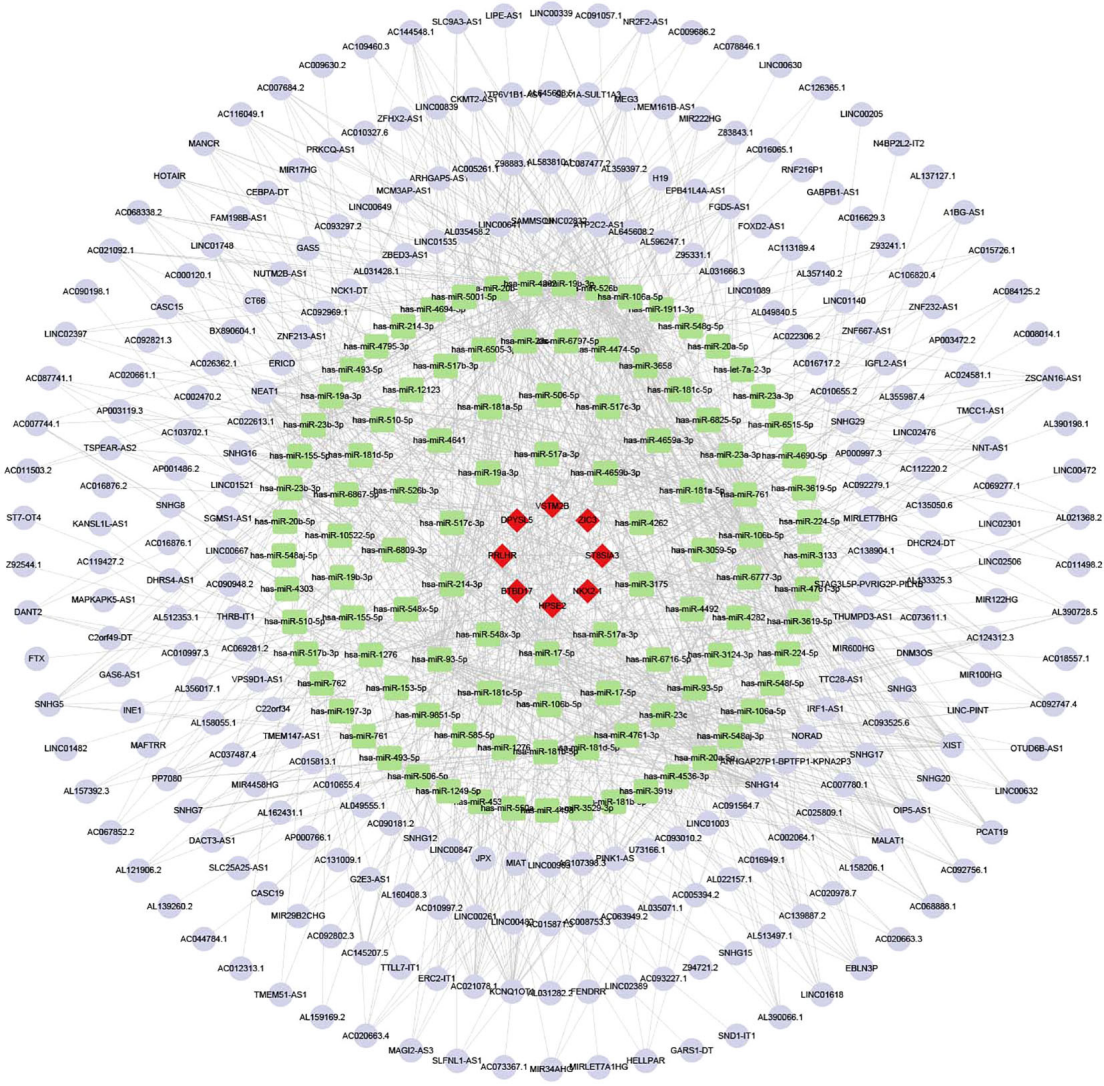
The ceRNA network. Red diamonds represent mRNA, green rectangles represent miRNA, and purple circles represent LncRNA.

In the ceRNA network built from TRMT6-based differential analysis, HPSE2 has the most nodes among mRNAs, hsa-miR-17-5p among miRNAs, and Lnc SNHG14 among lncRNAs (**Figure 9**).

## 4 Discussion

OC ranks as the eighth most common and the fifth most lethal malignant tumor globally. The incidence rate of OC is approximately 3.4%, with a mortality rate of about 4.7%. Each year, over 3 million women are diagnosed with the disease, and around 152,000 patients succumb to OC, posing a serious threat to women’s health and survival (23). In clinical practice, ovarian tumors are typically first detected via transvaginal ultrasound (TVS). However, TVS has certain limitations in terms of diagnostic accuracy, and the accuracy of diagnosis urgently requires improvement and optimization (24). Detecting serum biomarkers is a convenient, economical, and non-invasive method for predicting malignant tumors. Investigating the pathogenesis of OC and identifying more reliable biomarkers for the development of clinical predictive models can aid in the early detection of the disease and improve patient prognosis. In recent years, epigenetic research has garnered widespread attention, with approximately 170 types of RNA chemical modifications discovered across various RNAs, including both coding RNAs and non-coding RNAs (ncRNAs) (25–27). Among these RNA modifications, methylation modifications are the most common and extensively studied, accounting for over 60% of all RNA chemical modifications, including N6-methyladenosine (m6A), N1-methyladenosine (m1A), 5-methylcytosine (m5C), and N7-methylguanosine (m7G) (28, 29). Although research on the functions of RNA methylation modifications is still in its early stages, an increasing body of data indicates that dysregulation of RNA methylation plays a significant role in the development of various human diseases (30–32). Cancer cells often undergo genetic and/or epigenetic changes, which may accompany dysfunction of oncogenes or tumor suppressor genes (33, 34). As a prevalent form of post-transcriptional modification in epigenetics, RNA methylation plays a crucial role in the spatiotemporal regulation of gene expression (35, 36). Among these modifications, N6-methyladenosine (m6A) is the most extensively studied in the context of cancer (37, 38). Another significant post-transcriptional modification is N1-methyladenosine (m1A), which is primarily regulated by three types of enzymes: writers (TRMT6, TRMT10C, TRMT61A, TRMT61B), readers (YTHDC1, YTHDF1 - 3), and erasers (ALKBH1, ALKBH3). The investigation of m1A regulators in tumorigenesis remains in its early stages. Research indicates that TRMT6 is upregulated in hepatocellular carcinoma (HCC) tissues, and it correlates with poorer overall survival and recurrence-free survival rates (39). Additionally, the eraser ALKBH3 is overexpressed in lung cancer (LC) (40) and promotes cancer cell proliferation, migration, and invasion by inducing tRNA-derived small RNAs (41). Recent studies have revealed the correlation between m1A regulators and the mTOR and ErbB signaling pathways in gastrointestinal cancer (42). Research indicates that m1A regulators and methylation modification patterns significantly influence the evolving immune microenvironment during the development of OC (10, 43). The m1A-related phenotypes are associated with immune cell infiltration in the tumor microenvironment (TME), with distinct m1A patterns identified in immune desert, immune inflammation, and immune exclusion phenotypes (43). Furthermore, eight m1A regulators exhibit a positive correlation with activated mast cells, plasma cells, and M1 macrophages in abdominal aortic aneurysms. Notably, YTHDF3 has been demonstrated to promote M1 polarization of macrophages while inhibiting M2 polarization (44). However, the clinical significance of m1A regulators in OC remains poorly understood.

To address this issue, we utilized bioinformatics analysis to identify ten m1A regulators that exhibited differential expression between OC tissues and normal tissues, and were closely associated with prognosis. Subsequently, we validated the differential expression of the m1A regulator TRMT6 using qRT-PCR in collected OC tissues and normal tissues, which aligned with the results of our bioinformatics analysis. Given the association between TRMT6 overexpression and poor prognosis in OC, we further confirmed its statistically significant differential expression between OC and normal tissues through immunohistochemistry experiments. These experiments revealed that TRMT6 expression has a negative correlation with prognosis. Our research showed that the expression levels of TRMT6 correlate with ovarian cancer staging, but the expression levels of TRMT6 demonstrated no significant correlation with other clinicopathological features, such as lymph node metastasis or omental metastasis. This raises the question: through what mechanism does TRMT6 influence the prognosis of OC patients? Considering the current research indicating that m1A methylation modification significantly impacts the prognosis of OC and shapes the immune microenvironment, it is reasonable to explore whether this might be the key to understanding how TRMT6 affects the prognosis. It is essential to consider that TRMT6 may affect the prognosis of OC patients by participating in the regulation of tumor cell immune infiltration. Notably, our research findings suggest a negative correlation between TRMT6 expression and the infiltration of various tumor immune cells. Despite the weak correlation, statistical significance supports a potential association between TRMT6 and immune-infiltrating cells. The negative correlation (r < 0, p < 0.001) implies that TRMT6 may exert broad yet subtle regulatory effects on immune-infiltrating cells within the tumor microenvironment. This weak correlation may be attributed to several factors. 1. Large sample size. While a large sample size enhances statistical significance, the effect size may be diluted. 2. Multi-factorial regulation. The abundance of immune-infiltrating cells is co-regulated by multiple genes and pathways, limiting the contribution of a single gene, such as TRMT6. 3. Indirect effects. TRMT6 may indirectly influence immune cell infiltration by regulating other molecules, including RNA-modifying enzymes or immune-related factors. Moreover, the differences in enrichment scores of T cells gamma delta and Mast cells activated between the high and low expression groups also suggested that TRMT6 contributes to poor prognosis in OC patients by promoting tumor immune escape, rather than through conventional pathways like facilitating lymph node metastasis.

To delve into the mechanism by which TRMT6 influences the immune response in ovarian cancer, we conducted differential gene expression analysis based on the varying expression levels of TRMT6 and constructed a ceRNA network. As the most interconnected mRNA in the ceRNA network, the latest research showed that HPSE2 influenced tumor progression in multiple ways by facilitating interactions between tumors and host tissues. It created an ideal tumor microenvironment, promoting tumor growth, metastasis, and chemotherapy resistance (45). Given its multifaceted role in the tumor microenvironment, HPSE2 regulated these key traits, underscoring the need for HPSE2-targeted therapies (46). Besides the research on ovarian cancer, in the comprehensive analysis of the latest gastric cancer (GC) immune microenvironment-related ceRNA regulatory axis, researchers discovered that the LINC01133/miR-17-5p/PBLD axis played a crucial role in the development of GC (47). Researchers have discovered that the lncRNA SNHG14/hsa-miR-101-3p/KL/PLK1 regulatory axis plays a modulatory role in the immune microenvironment of lung adenocarcinoma (48). It is worth considering that TRMT6 may regulate the tumor immune microenvironment in ovarian cancer by interacting with HPSE, hsa-miR-17-5p, and lncRNA SNHG14. However, more in-depth fundamental experiments are needed to validate this conclusion.

The abnormal expression of the m1A regulator TRMT6 may influence patient prognosis by exerting indirect or synergistic effects within the tumor immune microenvironment (49). Pan-cancer analysis revealed that RNA methylation genes ALYREF, NSUN4, TRMT6, and YTHDF1 were associated with immune infiltration in the tumor microenvironment (49). GSEA and immune correlation analysis between different clusters suggested that m6A/m5C/m1A modification patterns played a significant role in the tumor microenvironment of gliomas, providing valuable information for anti-glioma immunotherapy (50). The study by Li et al. showed that the cluster subgroups and risk models of m6A/m5C/m1A regulatory genes were associated with poor prognosis and the immune microenvironment in hepatocellular carcinoma, potentially serving as a new tool for assessing the prognosis of hepatocellular carcinoma patients (51). Our study is the first to discover that the m1A regulator TRMT6 in OC may impact patient prognosis by promoting immune escape, which also provided a significant reference value for the immunotherapy of OC. In clinical practice, these study results may support developing TRMT6 blood-based testing to predict OC prognosis, and or assess immunotherapy response when combined with PD-1 testing. The TRMT6 gene worsens ovarian cancer prognosis by disrupting immune response pathways. Its major clinical implications are as follows: as a biomarker for prognosis, its expression levels can identify high-risk patients and guide personalized treatment; as a therapeutic target, inhibitors could reverse immune suppression, enhance immunotherapy efficacy, offering new ways to improve survival. However, the specific mechanisms underlying this influence require further experimental validation. Additionally, a more in-depth exploration of TRMT6’s role in OC treatment could contribute to the enhancement of targeted therapeutic strategies for OC.

## Data Availability

The datasets presented in this study can be found in online repositories. The names of the repository/repositories and accession number(s) can be found in the article/supplementary material.
